# Round the World
1Continuation of "Therapeutics of a Sea Voyage," March 1903, p. 1.


**Published:** 1903-09

**Authors:** R. Roxburgh

**Affiliations:** Honorary Physician, Weston-Super-Mare Hospital.


### ROUND THE WORLD.[1]

### A MEDICAL TRAVELLER'S NOTES.

BY

R. Roxburgh, M.B., F.R.C.S. Ed.,

*Honorary Physician, Weston-super-Mare Hospital.*

---

The British visitor to Australasia is immediately struck with the identity in all outward features of what is called "civilised life" in different parts of the world. Melbourne presents the

[1] Continuation of "Therapeutics of a Sea Voyage," March 1903, p. 1.

same imposing public buildings, massive offices, brilliant shops, blazing advertisements and irrational habits in dress to which we are accustomed at home. The business man goes to the city in torrid midsummer wearing a black coat and hat, as if it were sacrilege to consult his comfort and defy convention. These respectable cloth-covered backs often carry the addition of thousands of sessile flies; for the flies and the dust are the two *bêtes noirs* of Melbourne. It is difficult to believe that that great city with its 460,000 inhabitants, its magnificent streets and churches, its beautiful suburban gardens and dwelling-houses, was sixty-six years ago a mere hamlet of wooden bungalows inhabited by about 100 settlers. Such large urban populations in the colonies do not imply as in old countries overcrowded slums, filth, and moral and physical degeneracy. Melbourne covers an area of 254 square miles, and less than a sixth of the population live in the actual city; the others live in the suburbs, but everywhere is space, light and air. The poorest have something better than a mere "living wage," and society is far less strictly divided into layers than at home. A low death-rate corresponds with the better conditions of life.

Sydney, the capital of New South Wales, nearly equals Melbourne in population with its 430,000 inhabitants. An older city (it had its small and tardy beginnings in 1788), it was laid out less regularly than the capital of Victoria, and its streets have not that lavish breadth or mathematical straightness which characterise the more modern city. In the element of the picturesque, however, there can be no comparison. The beauty of Sydney Harbour has not been exaggerated. This blue inland sea, with its numerous bays and inlets, each deep enough to float the largest ships, and exquisitely wooded to the water's edge, is the pride of every citizen and the delight of every traveller. Sydney has two admirably appointed general hospitals, the Sydney and the Prince Alfred, and a flourishing medical school. When I was there in May of last year, the Public Health authorities were contending with bubonic plague. The principal medical officer of the State, Dr. Ashburton Thompson, and the able head of the Public Health Laboratory, Dr. Tidswell, kindly gave me access to the latter, and also to their reports on the disease and its propagation. They also introduced me to the Medical Superintendent of the "Coast Hospital" for infectious diseases. This last deserves special mention. It consists of an area of land several square miles in extent close to the sea, surrounded by high barriers, containing isolated groups of wooden buildings for the treatment of the different diseases. One group is for lepers, who are taken in for life, and is itself a miniature village. Others are for plague, scarlatina, small-pox, diphtheria, etc. It is eight miles from the city, being reached by a steam tramway. The buildings are on wild prairie ground sloping to the shore, and convalescents can live in the open air, and enjoy sea-bathing and rambling. In so large a space isolation is easily carried out, and the conditions are most favourable for recovery. If one or more wards become profoundly infected, they can be taken to pieces and burned, and new ones can be erected. Here is one great advantage of a country where land is cheap, and ample for municipal purposes. All the cases of plague in the wards at the time of my visit were mild and doing well, the bubos having been opened and dressed with iodoform gauze. One autopsy I saw at the General Hospital—a Chinaman, who had died twenty-four hours after infection, an extremely septic case. Here it may be mentioned that the Chinese, who have to pay a poll-tax of £100 to enter Australia, and are hated by white labourers for the small pay they are willing to accept and their extraordinary economy in living, are, according to the ward sisters of the General Hospital, excellent patients—docile, cleanly and obliging.

A day was advantageously spent in the pathological museum of Sydney University, and another morning I was invited by Mr. MacCormick, of the Prince Alfred Hospital, one of the ablest surgeons in Australasia, to attend some operations. He performed five major operations, including removal of an upper-jaw, and of a sarcoma of the anterior triangle, between the hours of 8 a.m. and 1 p.m., with admirable dexterity, and without the slightest sign of fatigue. In the equipment of the theatre no aseptic appliance was wanting. Here let me mention the exceeding kindness extended to a professional brother by all colonial medical men.

Though I have referred to Sydney at this point, I did not go there immediately from Melbourne, but sailed first from Melbourne to Dunedin, a voyage of about 1,520 miles. I passed rather rapidly through New Zealand from south to north, arriving at Auckland after five weeks of walking, coaching and sailing in that most beautiful country. Nothing of much interest from a medical standpoint came in my way. The University of New Zealand, which examines and grants degrees, consists of the three colleges of Dunedin, Christchurch and Auckland. The medical school of the country is connected with the Dunedin College. Of the hospitals of New Zealand, forty-three in number, one half are incorporated institutions under the government of trustees; the other half are managed by elective "Hospital District Boards," who have also to contribute to the maintenance of the incorporated hospitals. They are supported (1) by voluntary subscriptions and bequests, (2) by rents and profits from lands vested in the boards, (3) by payments from patients, (4) by contributions from local authorities, and (5) by Government. The last item averages two-fifths of the whole, the voluntary contributions being about one-twelfth of the whole. Whether this system of State control works entirely well I am unable to say.

The death-rate of New Zealand is very low in comparison with that of European countries. It was 9.43 per 1,000 in the year 1900, that of Great Britain being 18.4, of France 21.1, of Germany 21.5, and of Austria 25.4. In the Australian Commonwealth, including Tasmania, it was in 1899 12.85. The deaths from phthisis in New Zealand in 1900 were 7.56 per 10,000. In England and Wales the lowest rate for many years was reached in 1896, when it was 13.07. Seeing that many sufferers from phthisis go to New Zealand yearly for their health, the death-rate shows how favourable that country is as a place of residence for phthisical patients. I observed posted up at every railway station, post-office, and public place, a clearly expressed, concise notice, regarding the genesis and prevention of tuberculosis, issued by the Government Public

Health authority, and calling on the public to assist the State and the medical profession in stamping out the disease. It was printed in such type as to arrest the attention of any one who had his eyes open. Why should not the same sensible measure be adopted by our Local Government Board ? We have endless advertisements of quack remedies on every hoarding, but that which really affects the well-being of mankind is left undone. A government of socialistic type is not without its advantages. In New Zealand the railways are the property of the State, which can use its advertisement spaces as it will.

The climate of New Zealand is more equable than that of Great Britain, the mean for the whole colony being in spring 55° F., in summer 63°, in autumn 57°, and in winter 48°. London is 7° colder than the north, and 4° than the south island. There is an immense difference in the rain fall between the western and eastern coasts. In the south island the great longitudinal chain of the Southern Alps, parallel with the west coast, precipitate the rain on that coast to an extent five times greater than on the east. The eastern districts are the driest, the great Canterbury Plain ending at Christchurch in the south island, and the vicinity of Napier in the north island, being almost ideal in climate as well as productiveness.

This is hardly the place to dwell on the magnificence of the scenery, or the wonderful flora and fauna of New Zealand, which are a perpetual source of interest and surprise. Switzerland, England and Scotland are all to be found there. The Southern Alps have an average height of 8,000 feet, the highest, Mount Cook, reaching 12,349 feet. A large proportion of these mountains is still unexplored. The inland lakes and the coast fiords or "sounds" are majestic.

The hot springs of the north island are found over an area of comparatively recent volcanic action, extending from north to south a distance of 300 miles. There are about seventy well-known medicinal springs — saline, alkaline, silicious, sulphurous, and acid, but not chalybeate, besides many remarkable geysers and other phenomena of great interest. Rotorua is the centre for these, and most of the European medicinal baths have their counterparts there. The visit of the Prince and Princess of Wales to that district had the good effect of stimulating the local authorities to the improvement of the appointments of these baths.

From Auckland I visited the Tongan, Samoan and Fijian groups of the Polynesian Archipelago, a novel and charming experience. In Tonga Tabu, the principal of the "Friendly Islands," Dr. McLennan, the only European medical man, was most hospitable. The greatest difficulties of his practice, he said, were the native habit of consulting Tongan "medicine men" and dosing themselves with poisonous concoctions, and, secondly, the impossibility of obtaining milk. There are no cattle on the islands, and if there were, the natives would prefer the milk of the green cocoanut to which they are used.

The national diet consists of bread-fruit, taro, yam, banana, with occasional indulgence in roast pig or fish. With Dr. McLennan I paid a professional visit to the young Queen of Tonga, who seemed to be suffering from tuberculosis of the peritoneum: she died a few months afterwards. The physical development of the Tongan race is an argument for vegetarianism. More magnificent men than these tall copper-coloured, half-naked denizens of the palm-groves I never saw: they are both handsome in feature and most dignified in carriage, and their musculature would stir the enthusiasm of a sculptor or anatomist.

In all the islands of the South Pacific the *filaria* is a common parasite, and elephantiasis is frequent. In Suva, the capital of Fiji, Dr. Lynch took me over the native hospital, and showed me several cases where elephantiasis scroti of enormous dimensions—weighing sometimes 80 to 90 lbs.—had been successfully removed. The operation is not difficult, and the relief to the patient can be imagined. In Fiji also I saw many instances of yaws (Frambœsia), in every stage of the complaint, fungoid, ulcerative, and "tertiary" (periostitic and destructive).

Sometimes a sucking-child, with typical ulceration of the mouth, had communicated a similar ulcer to the maternal nipple and areola. But the Fijian mothers are quite content: they believe it beneficial to the child to have the disease in infancy, and in most cases an early attack does confer future immunity. Mr. Jonathan Hutchinson's view, that yaws is a manifestation of lues, is not shared by the profession in Fiji, who are convinced that, though offering many analogies to syphilis, such as that potassium iodide and mercury are specifics for both, it is an entirely separate pathological entity, and is not of venereal origin. Syphilis, indeed, seems to be unknown in Fiji. This is the opinion of Dr. Corney, the highest and most experienced authority in that part of the world.

Samoa, or the "Navigator Islands," lie between 13½° and 14° latitude south of the equator: Fiji is between 15° and 20°: Tonga between 18° and 22°. Thus these island groups lie well within the tropic of Capricorn, and their vegetation is correspondingly luxuriant. From October to May they are visited by tropical rains, so that the climate is both hot and moist. From May to October, the dry season, is the most agreeable. The climate of Fiji, though it has a mean temperature of 80° F., is in the dry months most pleasant, and the scenery, combining rugged red peaks of igneous rock with glorious vegetation, is peculiarly lovely. There is indeed an indescribable fascination about all these islands, with their fringing and barrier-reefs of coral, on which the Pacific billows break, their quiet and many-tinted lagoons, their palms, bread-fruit trees, casuarinas, bananas and other fine trees, their brilliant dracænas and crotons, frangipanni and hibiscus flowers, and the green carpet of the sensitive plant, while the gentle and open manners of the natives are in happy contrast to the more stand-off and self-sufficient habits of intercourse which characterise Anglo-Saxons.

After spending part of March and most of April in the South Seas, I returned to Sydney, and sailed thence for Japan, on the 8th of May. The fine Clyde-built Japanese steamer, *Kumano Maru*, coasted along the east side of Queensland, passed through Torres Straits, by New Guinea, through the Banda Sea, past Celebes and the other Dutch spice Islands, called at Manila in the Philippines, and at Hong Kong, touched at Nagasaki and Kobe on the "inland sea," and finally reached Yokohama, thirty days after leaving Sydney.

I must not leave the subject of Japan without referring to the great kindness of Dr. Miller, of Kobe, and his colleague Dr. Thornycroft.

Space forbids me to enter on the popular subject of Japan, with which it would be easy to fill the *Journal*. I may mention, however, that during a stay of five weeks there I visited several medical schools, and found every modern appliance for the teaching of the medical sciences. Japanese is used as the medium of instruction, but technical terms are generally expressed in German, owing, no doubt, to the fact that so many Japanese students are trained at German medical schools. Most of the medical literature also which I saw was German. This is in contrast with the general custom, which is to use English alongside of Japanese for all official notices, such as those at post-offices and railway stations, and often for unofficial advertisements.

Japan is volcanic in origin, and earthquakes are common. This also accounts for the numerous hot springs and *solfataras* scattered throughout the country. Bathing is a passion with the Japanese: they are accustomed to use water at a much higher temperature than we should—110° to 120° F.; but these very hot baths do not weaken or cause re-action, and are found to be most refreshing after a long day's walking or riding. A very curious sight is the Spa Kusatsu, a town 3,800 feet above sea-level, which contains a leper settlement, and is also largely resorted to for the treatment of syphilis, rheumatism, and gout. I called on the resident doctor, but found he knew neither English nor German. He, however, escorted us through the place, and my guide interpreted. He took us to the chief public bath, where about twenty-five men systematically bathe in scalding water containing 3.176 per cent. of sulphuric acid, 6.476 per cent. of alum, and traces of arsenic and sodium. They go through this penance at 5 a.m., 9 a.m., 2 p.m. and 5 p.m., and are summoned at these hours by the blowing of a horn. The water being from 113° to 128° F., they first spend a quarter of an hour in splashing it up with long boards to a rhythmic chant, partly to cool it, partly to get into a perspiration; they then baste their heads a hundred times with wooden ladles, while they kneel round the edges of the tank. At a given moment the bath-master shouts "In"! and they descend screwing up their faces with pain, till they are immersed up to the chin. The bath-master calls—"One minute," and they respond with a hoarse cheer; "two minutes," "three minutes," and "Out!" They crawl out, red and half-boiled, and then dress and leave, to go through it all again in four hours. The doctor informed me that he had never seen any benefit to leprosy from these baths, but the lepers had unconquerable faith in them. The patients whom I saw in the public bath seemed for the most part to have rupia or macular syphilides. It is difficult to see how so irritating a method of treatment could be of service in these cases.

Leaving Yokohama on 15th July, I crossed the Pacific *via* Honolulu, and was in San Francisco on the 30th. The climate of Los Angeles and San Diego, at the southern extremity of California, is almost ideally perfect, for though the sun is intensely strong, the atmosphere is remarkably dry, and a cool refreshing breeze blows in continually from the ocean. The great central fruit-growing districts of California are very trying in summer from the fierceness of the sun's rays, unmodified by the influence of the sea, and fruit-growers there are often glad to take flight in midsummer to Santa Catalina or Los Angeles.

After some weeks in California I returned to San Francisco, thence by rail to Vancouver, and crossed the Rockies and Selkirks, by the Canadian Pacific Railway, which I left at Fort William, to steam down Lake Superior and Huron. I spent some time with friends in Nova Scotia, and returned to England *via* New York and Liverpool, the whole voyage from Plymouth round the world to Liverpool having occupied exactly a year and five days.

